# Delay-Tolerance-Based Mobile Data Offloading Using Deep Reinforcement Learning

**DOI:** 10.3390/s19071674

**Published:** 2019-04-08

**Authors:** Daisuke Mochizuki, Yu Abiko, Takato Saito, Daizo Ikeda, Hiroshi Mineno

**Affiliations:** 1 Graduate School of Integrated Science and Technology, Shizuoka University, 3-5-1 Johoku, Naka-ku, Hamamatsu-shi, Shizuoka 432-8011, Japan; abiko@minelab.jp; 2 NTT DOCOMO, INC., Urban Sensing Research Group, Research Laboratories, 3-6 Hikari-no-oka, Yokosuka-shi, Kanagawa 239-8536, Japan; takato.saitou.bu@nttdocomo.com (T.S.); ikedad@nttdocomo.com (D.I.)

**Keywords:** mobile data offloading, reinforcement learning, delay tolerant

## Abstract

The demand for mobile data communication has been increasing owing to the diversification of its purposes and the increase in the number of mobile devices accessing mobile networks. Users are experiencing a degradation in communication quality due to mobile network congestion. Therefore, improving the bandwidth utilization efficiency of cellular infrastructure is crucial. We previously proposed a mobile data offloading protocol (MDOP) for improving the bandwidth utilization efficiency. Although this method balances a load of evolved node B by taking into consideration the content delay tolerance, accurately balancing the load is challenging. In this paper, we apply deep reinforcement learning to MDOP to solve the temporal locality of a traffic. Moreover, we examine and evaluate the concrete processing while considering a delay tolerance. A comparison of the proposed method and bandwidth utilization efficiency of MDOP showed that the proposed method reduced the network traffic in excess of the control target value by 35% as compared with the MDOP. Furthermore, the proposed method improved the data transmission ratio by the delay tolerance range. Consequently, the proposed method improved the bandwidth utilization efficiency by learning how to provide the bandwidth to the user equipment when MDOP cannot be used to appropriately balance a load.

## 1. Introduction

In recent years, the types of mobile data have diversified due to improvements in mobile device performance [1]. Furthermore, Internet-of-things (IoT) devices have been widely used. IoT devices upload data, such as images and sensor data and movies, which are frequently aggregated [2]. The upload data is increasing owing to the increase in the number of IoT devices. Not only is IoT device traffic expected to increase sharply, but machine-to-machine (M2M) traffic is also expected to increase. In M2M communication, devices that operate individually without human operation are autonomously controlled. The communication demand of mobile data traffic may increase further. Furthermore, mobile data traffic has a characteristic that it is biased towards specific times and in certain areas, such as commuting time and at stations, respectively [3]. This characteristic of mobile data traffic decreases the bandwidth utilization efficiency of cellular infrastructure. In order to increase the bandwidth utilization efficiency, it is desirable to accommodate the traffic while maintaining the load of the cellular infrastructure, such as evolved node B (eNB), within a certain allowable range. However, if the cellular infrastructure is provided while considering the characteristics of the demand naively, a problem occurs: the utilization rate of the cellular infrastructure and the traffic accommodation efficiency decreases in areas where the demand for communication is low or in that particular time zone. In order to distribute the demand, mobile communication carriers have worked on establishing Wi-Fi spots, although it is challenging to install Wi-Fi spots based on the demand. 

To balance the load, a method is used that automatically sets an optimum network according to a dynamic change in mobile data traffic [4]. In another method, a load of eNB is distributed by controlling the transmission rate of the user equipment (UE) [5,6]. This load balancing method comprising mobile data offloading is expected to reduce cellular network congestion in a cost-effective manner [7]. As a load distribution method that takes into consideration the locality of the communication demand, we previously proposed the use of a mobile data offloading protocol (MDOP) [5,6], which is a method that is focused on the content delay tolerance [8,9]. In this method, the transmission rate of the UE is controlled in three ways: time, place, and link. It has been confirmed that time-wise offloading of MDOP can reduce the time-wise locality; however, there is scope for improving the load-balancing performance in various situations. Although the load is properly balanced in this method by controlling the transmission rate of the UE under different conditions of the topology structure, the traffic model of the UE, and eNB loads, it is difficult to always maximize the performance depending on the circumstances. 

In addition to the aforementioned method, there exists a transmission-rate control model focused on content [10]. In [10], the bandwidth is equalized for UE with real-time content that allows short delays, such as video data. Moreover, in [11], the load is distributed by focusing on the UE’s content type, load status, and contract information in order to suppress the overload condition. However, the network environment is complicated because of the expansion of eNB for load balancing and the diversification of content owing to the improvement in the performance of mobile devices. Hence, it is difficult for the transmission-rate control model formulated as in [10,11] to always provide the appropriate control. Therefore, it is necessary to construct a transmission rate control model dynamically according to the network environment for providing proper transmission rate control in a complicated network environment. Furthermore, [12] proposes a transmission rate control that focuses on devices that generate content. In [12], the bandwidth of M2M communication is allocated by taking into consideration the degradation of the quality of service (QoS) between the UE. The transmission rate control with emphasis on M2M devices is crucial as the demand for M2M communication is expected to increase rapidly. In contrast, the real-time characteristics are necessary for autonomous driving content, which is a type of M2M communication. Thus, focusing on content delay tolerance can improve the performance and quality of user experience.

On the other hand, reinforcement learning has been applied in various fields in recent years [13]. Reinforcement learning is a learning method in which an agent optimizes the action through repeated trial and error [14]. It is confirmed that reinforcement learning can obtain a higher score than humans in board games by performing appropriate actions based on acquired experience [15,16]. The method used for handling various situations based on the acquired experience may be applied to satisfy various communication demands [17,18,19]. For example, [17] comprises the use of reinforcement learning for power control and rate adaptation in the downlink of a network. In addition, in [18], deep reinforcement learning is applied for cost and energy aware multi flow. Although it has been assumed in previous studies related to LAN offloading problems from mobility users that the mobility pattern of the UE is known in advance, [18] has become unnecessary. In recent years, reinforcement learning has been applied in cellular networks, and the obtained results are shown in [17,18,19]. Therefore, there is also a possibility that reinforcement learning can be used to resolve the locality of the mobile data traffic demand.

In this paper, we propose and evaluate a delay-tolerance-based mobile data offloading method using deep reinforcement learning for improving the bandwidth utilization efficiency of cellular infrastructure and solving the traffic demand locality. If an agent can learn how to balance the load properly, considering various situations such as traffic model of the UE, topology structure, and eNB loads, proper balancing can be achieved under conditions for which time-wise offloading of MDOP cannot balance the load properly. Our method performs transmission rate control by determining the priority for allocating the bandwidth to the UE as per the quality of service level (QoS level) based on the content delay tolerance of the UE and the conditions of the eNB such as the load.

## 2. Delay-Tolerance-Based Mobile Data Offloading Using Deep Reinforcement Learning

### 2.1. Overview

In the proposed method, we apply deep reinforcement learning to load balancing in order to improve the bandwidth utilization efficiency by enabling appropriate transmission rate control even in situations where appropriate load balancing control is difficult in time-wise offloading of MDOP. We expect that improve the efficiency of accommodating mobile data to the communication infrastructure by balancing the eNB load. The time-wise offloading of MDOP offloads the traffic by allocating bandwidth with a focus on delay tolerance of content and improves the bandwidth utilization efficiency. On the other hand, there is a problem that it is difficult to control appropriately with rule-based control in the diversified network environment. To get an approximate solution of appropriate control and improve the bandwidth utilization efficiency in the various network environments, we use deep reinforcement learning for mobile data offloading. Our method applies to the time-wise offloading of MDOP as a transmission rate control model that takes into consideration the delay tolerance. The details of the MDOP are described in Section 2.2. Although the time-wise offloading of MDOP can reduce the time-wise locality, there is scope for improving the bandwidth utilization efficiency by focusing on the characteristics of the mobile data traffic such as delay tolerance and content size. Because the time-wise offloading of MDOP transmission rate control is performed using a model formulated based on the traffic model of the UE, topology structure, and eNB loads, it is not always possible to properly control the situation of massive mobile data traffic. Our method dynamically constructs a transmission rate control model according to the mobile data traffic characteristics using deep reinforcement learning. Therefore, this method enhances the bandwidth utilization efficiency.

**Figure 1:**
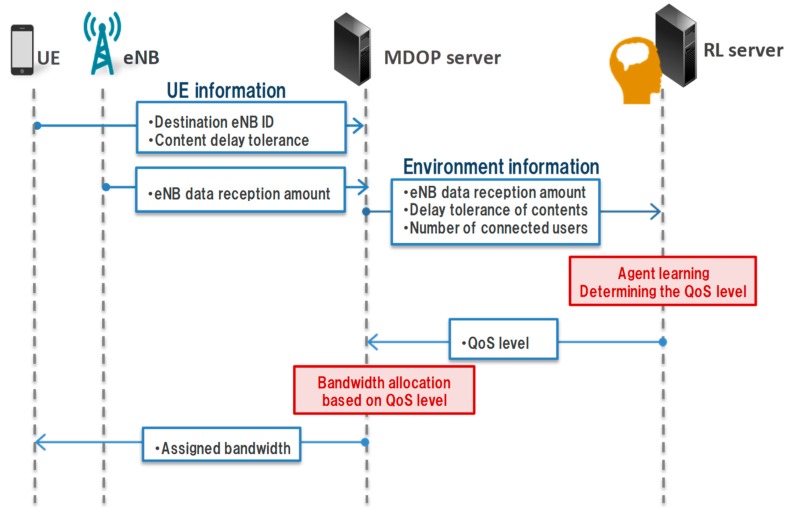
Learning flow of the proposed method.

A learning flow of our method is presented in Figure 1. The reinforcement learning server (RL server) is the agent that performs the learning. The RL server learns based on environment information, which is collected by the MDOP server. The environment information consists of the eNB data reception amount and UE information. The UE information includes the content delay tolerance, the remaining amount of content, and the destination eNB identification (eNB ID).

The RL server determines the priority of the bandwidth allocated to the UE according to the environment information. We define this priority as the QoS level. Subsequently, the MDOP server allocates the bandwidth to the UE based on the determined QoS level. The UE then sends the data according to the allocated bandwidth based on the QoS level. Finally, the RL server learns the priority for allocating the bandwidth from the eNB’s load fluctuation caused by the sending data of the UE and constructs a transmission rate control model.

### 2.2. Mobile Data Offloading Protocol

**Figure 2:**
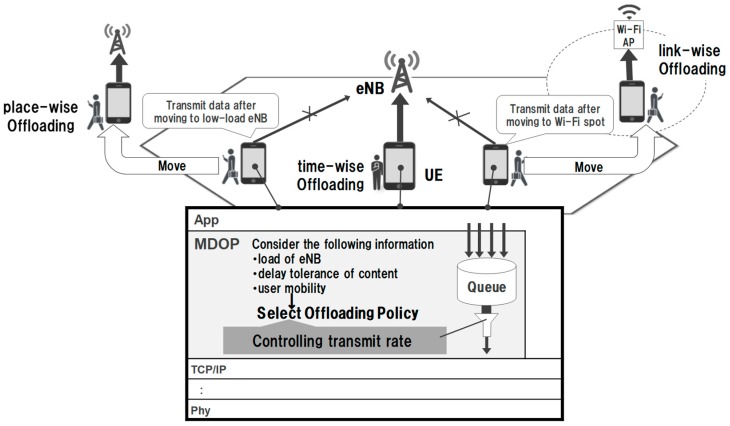
Overview of the mobile data offloading protocol (MDOP).

Previously, we proposed an MDOP that balances the load of the eNB while taking into consideration the locality of the demand [5,6]. Figure 2 presents an overview of MDOP. MDOP is implemented by middleware located in the lower layer of the application layer. It realizes mobile data offloading by controlling the transmission rate in consideration of the delay tolerance when transmitting and receiving corresponding applications. MDOP has three methods of controlling the transmission rate as offloading policy. First, the time-wise offloading solves the time-wise locality by delaying the communication when the eNB has a high load. Second, place-wise offloading solves the regional locality by delaying the communication of the UE connected to a high-load eNB until it connects to a low-load eNB. Finally, link-wise offloading is used to reduce the traffic on a mobile data channel by delaying the communication of the UE until a connection to a Wi-Fi access point is established. MDOP selects the offloading policy from these three methods according to the state of the delay tolerance of the data and the state of the UE and eNB. MDOP then executes transmission rate control using the selected policy.

In MDOP, an MDOP server periodically collects content information from UEs, load information from eNB, and performs band allocation. When an MDOP server notifies the UE of the allocated band, the UE can transmit content with the allocated band. When band allocation is performed, the maximum band is allocated to UE if the content does not have delay tolerance. Also, if the content has delay tolerance, the bandwidth is divided equally. 

MDOP controls the transmission rate to accommodate a load within the control target value. We defined an ideal load as the control target value. The ideal load is set in order to prevent the occurrence of situations where packet loss and the allowable amount of eNB is exceeded when burst traffic occurs. MDOP reduces the locality of the demand by accommodating the eNB load within the ideal load to smooth the eNB load. When the content delay tolerance is exceeded, the content is transmitted at the maximum transmission rate without taking into consideration the ideal load.

Similar to the problem of the previous MDOP, the delay tolerance of the content is not taken into consideration. Therefore, the short delay tolerance content has the possibility to exceed delay tolerance because the same control is applied to all the content regardless of the delay tolerance. The proposed method focuses on the delay tolerance of the content and clarifying the priority to assign the bandwidth. The proposed method can balance the time-wise concentrated load by realizing the delay-tolerance-based bandwidth allocated method of UE. By using deep reinforcement learning, it is possible to determine the appropriate priority of bandwidth allocation under various situations when deciding the priority.

### 2.3. Transmission Control by Deep Reinforcement Learning

We determine the transmission control of the UE by using deep reinforcement learning, and the RL server learns using a double deep Q-network (DDQN) [20]. DDQN is a reinforcement learning method that uses deep learning with Q-learning [21]. Q-learning is focused on maximizing a function Q(s,a). Q(s,a) represents the value of action a taken in state s. Q-learning updates $Q\left(s_{t},a_{t}\right)$ at time t as follows:

(1) $$Q\left(s_{t},a_{t}\right)\leftarrow Q\left(s_{t},a_{t}\right)+\alpha \left(r+\gamma \underset{a^{\prime }}{\max}Q(s_{t+1},a^{\prime })-Q\left(s_{t},a_{t}\right)\right)$$

where α is the learning rate, and γ is the discount factor. Each parameter is defined as 0≤α, γ≤1. Furthermore, Q-learning selects the action with the highest Q-value. If we use Q-learning to perform learning, it is necessary to prepare a table function Q(s,a) which is a combination of all states s and actions a in advance. However, it is difficult to prepare this table function because there are innumerable situations in mobile networks. In the case of such a problem, deep Q-network (DQN) is used, which approximates Q(s,a) with a neural network [15]. Although a method for approximating Q(s,a) with a neural network has been previously proposed, it was known that the learning diverges as the number of parameters increases. Because the correlation between the data is high, the policy of the selecting action is changed significantly on updating Q(s,a). In order to prevent the divergence of the learning, DQN uses experience replay and neural fitted Q. Experience replay stabilizes the learning by using randomly sampled states and actions of the past. Neural-fitted Q fixes parameters to be approximated with a neural network for stabilizing the learning. DQN updates the Q value using the loss function L(θ) as follows:

(2) $$L\left(\theta \right)\;=\;\frac{1}{2}\left(r+\gamma \underset{a^{\prime }}{\max}Q(s_{t+1},a^{\prime }\right)-Q\left(s_{t},a_{t}\right){)}^{2}$$

However, DQN overestimates the action because Q(s,a) selection and Q(s,a) estimation models are the same. In contrast, the DDQN uses different models for the selection and estimation. Thus, the overestimation of the action in the case of DDQN is reduced compared with that in the case of DQN. In addition, we apply dueling-network architecture to DDQN [22]. Dueling-network represents Q(s,a) as follows:

(3) Q(s,a) = V(s)+A(s,a)

V(s) is a state value function and A(s,a) is a state-dependent action advantage function. V(s) shows the worth of a particular state s, and A(s,a) represents a relative measure of the importance of each action a. On using dueling-network architecture, it is possible to directly express a value of the state without using the value of the action. Furthermore, it is confirmed that the convergence of the state value occurs faster as a result. Hence, we use DDQN and dueling-network architecture to construct a transmission rate control model. 

Defining the states, actions, and reward is essential for learning using deep reinforcement learning. Table 1 lists the learning parameters of the proposed method at t. In our method, we construct a transmission rate control model for each eNB. Additionally, when multiple UE exists in the same eNB, the MDOP server aggregates UE information from each UE. Besides, the RL server determines the QoS level for each UE and performs bandwidth allocation. We define the environment information as state s. The RL server periodically receives environment information from the MDOP server. The environment information includes UE information and connected eNB information. The UE information includes the remaining amount of content (*content_ra_*), the content delay tolerance (*content_dt_*), and the destination eNB ID. Moreover, the eNB information is that gathered by the MDOP server, such as the available bandwidth and current load of the eNB. 

As the input parameter of the RL server, the UE information consists of the control target UE and the UE connecting to the same eNB. Although the QoS level can be determined based only on $content_{ra}$ and $content_{dt}$ of the control target UE, we introduce relative information to assign the UE priority in detail. The relative information to be introduced includes the maximum, median, and minimum values of $content_{ra}$ and $content_{dt}$. We can expect that the RL server decides the QoS level to be assigned to control the target UE in consideration of the UE connected to the same eNB by sending the relative information of the UE to the RL server. We also define the current time as a learning parameter, because it is used to evaluate the action of the RL server while taking the time information into consideration. Furthermore, we define all the parameters as one parameter in order to avoid learning that not converging too many states although a parameter can be defined for each QoS level.

We define the priority of the bandwidth allocated to the UE as action a. There are five QoS levels. The bandwidths are allocated in the order of QoS1 to QoS3. Furthermore, we assign QoS0 to a non-MDOP UE and an MDOP UE that has content that exceeds the delay tolerance. In MDOP, the content is sent at the maximum transmission rate when the delay tolerance of the content is exceeded. Moreover, we assign QoS4 to the UE that does not need to allocate bandwidth. Although as the learning progresses, the RL server can learn to avoid allocating a bandwidth to a specific UE, and the learning will become efficient if this control is given to the RL server as an action. Hence, the RL server assigns one of QoS1 to QoS4 to the UE. The role of the RL server is to determine action a using collected UE and eNB information via the MDOP server, which is the priority of the bandwidth to be allocated to the UE. The actual transmission rate is determined by the MDOP server based on the QoS level assigned by the RL server. The MDOP server allocates the bandwidth in order from the UE with a high QoS level. 

Finally, the reward r is derived by comparing the current load $L_{t+1}$ and the ideal load $L_{ideal}$ for each eNB. $L_{ideal}$ is constant regardless of time. Action a is evaluated using the reward function. The RL server then learns based on this evaluation result. A reward function of the proposed method is shown in Algorithm 1. First, we compare $L_{t+1}$ with $L_{ideal}$ in order to evaluate action a in terms of whether the load balancing of eNB is achieved. Reward r is a positive reward when $L_{t+1}$ is lower than $L_{ideal}$. In contrast, reward r is a negative reward when $L_{t+1}$ exceeds $L_{ideal}$. Thus, we set that the positive reward as +1 and the negative reward as the value determined according to the difference of $L_{t+1}$ and $L_{ideal}$. If the negative reward value is a fixed discrete value, action a is evaluated in the same manner regardless of the difference of $L_{t+1}$ and $L_{ideal}$ when $L_{t+1}$ exceeds $L_{ideal}$. Therefore, we define a negative reward using continuous values to avoid such an evaluation. However, despite action a, $L_{t+1}$ sometimes exceeds $L_{ideal}$. In this situation, the RL server should not allocate the bandwidth to the UE. Thus, reward r is zero value when the RL server outputs QoS4 as action a in this situation. Furthermore, we weight reward r by the elapsed time. In the proposed method, it is preferable to delay the mobile data communication and maintain the eNB load within $L_{ideal}$. However, the control becomes increasingly difficult as the elapsed time increases in the case of delaying and controlling the mobile data communication. Hence, in Algorithm 1, the longer the control elapsed time, the greater the positive reward when the RL server gets a reward. Conversely, the shorter the control elapsed time, the greater the negative reward. The episode end time is set by assuming that the RL server periodically controls within a certain time range, such as 1 day. Accordingly, the RL server can balance the load by considering the content delay tolerance while not exceeding $L_{ideal}$, since reinforcement learning learns the action to maximize the reward.
**Algorithm 1** Reward function of the proposed method1Current load of connecting eNB: $L_{t+1}$
2Ideal load that is control target value: $L_{ideal}$
3Available bandwidth of connecting eNB: ABW
4Select action at time t: $a_{t}$
5Episode end time: $t_{end}$
6Normalization variable: β
7**if**$L_{t+1}\;\leq L_{ideal}$**then**8 $r\leftarrow 1+\frac{t}{\beta }$
9**else**10 **if**
ABW = 0 and $a_{t}\;=\;4$
**then**11  r←0
12 **else**13  $r\leftarrow -(\frac{L_{t+1}-\;L_{ideal}}{L_{ideal}}+\frac{t_{end}-t}{\beta }$)14 **end if**
15**end if**16**return**r


## 3. Evaluation and Discussion

### 3.1. Evaluation Condition

**Figure 3:**
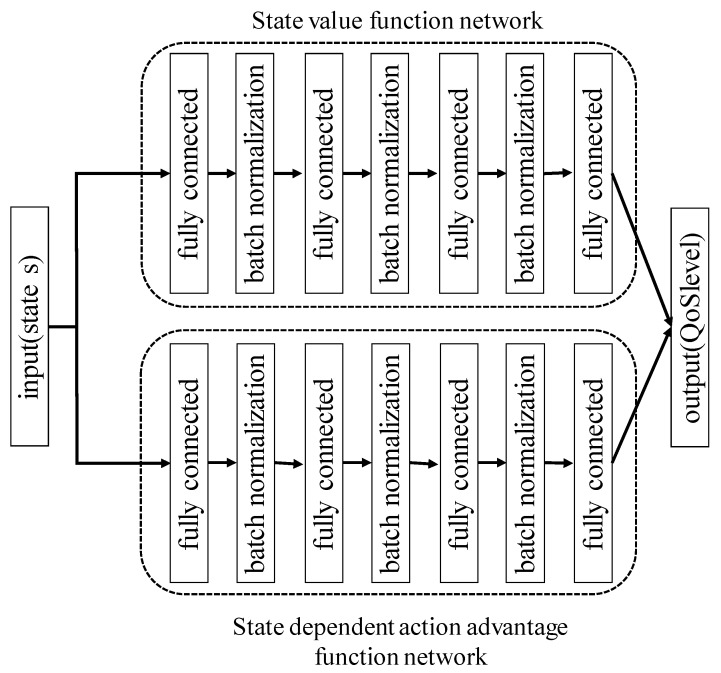
Network architecture of the proposed method.

We performed two evaluations to confirm the performance of the proposed method. Firstly, we evaluated whether the state, action, and reward are appropriate as definitions for the RL server to achieve the goal, which is to eliminate the temporal locality, in Section 3.2. We then evaluated the cellular infrastructure utilization efficiency of the proposed method in comparison with the no-control and the time-wise offloading of MDOP in Section 3.3. In this evaluation, it is necessary to determine the network structure and hyper-parameter of the proposed method. Figure 3 shows the network architecture of the proposed method. This network architecture consists of an input layer, four-hidden layers, and an output layer. We set the input to states in Table 1 and the output to QoS level. Furthermore, the network is divided such that we can output the state value function and state-dependent action advantage function separately because we apply the dueling-network architecture. The output of the state value function is 1 because the state value function represents the worth of a particular state s. Also, the output of the state-dependent action advantage function is 4 because this function represents a relative measure of the importance of 4 QoS levels. In addition, we use batch normalization [23] for obtaining an accelerated learning. The state value function network has unit numbers of 12-400-200-100-1 in order from the input layer. Similarly, the state-dependent action advantage function network has unit numbers of 12-400-200-100-4. This network outputs action after layers the state value function and state-dependent action advantage function are concatenated to represent Q(s,a) following Equation (3). The output is 4 because we defined there are 4 QoS levels for MDOP UE. The activation function is a hyperbolic tangent (tanh). The discount factor γ and learning rate α, which are the parameters of the Q-learning, are 0.96 and 0.01, respectively. In addition, we set the normalization variable β as $10^{3}$. The Q-learning selects actions such that Q(s,a) becomes the maximum value, which can imply overfitting. In order to avoid overfitting, we use a linear decay ϵ-greedy policy that selects a random action with probability ϵ and follows an action of the maximum value with probability 1 − *ϵ*. The linear decay ε-greedy policy decreases ε linearly. We decreased ε linearly from 1 to 0.01.

In this evaluation, we used CPU i7-6700k for learning. Besides, we used Scenargie, which is a network simulator, to accurately reproduce the long-term evolution (LTE) environment [24]. Furthermore, we determined the cellular network parameters to make scenarios according to 3rd generation partnership project (3GPP) and next generation mobile networks (NGMN) [25,26]. Table 2 shows the network environment model. We later present the details of the scenario and topology used for each evaluation.

### 3.2. Basic Evaluation

**Figure 4:**
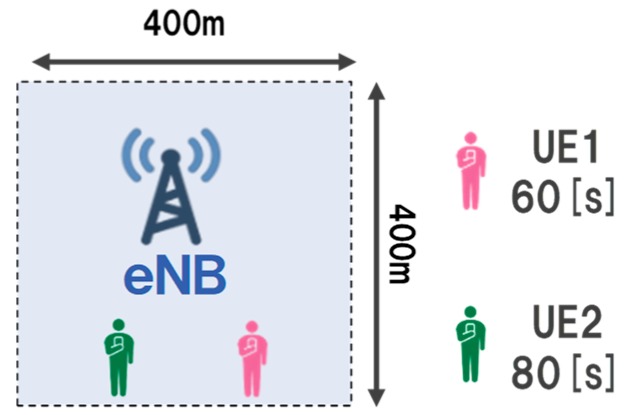
Topology of basic evaluation.

First, we performed a basic evaluation to confirm that the proposed method can perform appropriate control in the scenario where the priority to be allocated is explicit. Table 3 shows the parameters of the basic evaluation scenario. Moreover, Figure 4 presents the topology of the basic evaluation. In the basic evaluation, the number of UE is 2, and the content delay tolerance held by each is 60 s and 80 s. The content size is set such that it can be sent when allocating the bandwidth preferentially to the UE with a short delay tolerance. Thus, it is ideal that the RL server learns the control to assign a high priority to the UE that has the delay tolerant 60 s content. We set the episode end time $t_{end}$ as 100 in Algorithm 1 as the simulation time is 100 s. We set the maximum send rate of the UE as 1000 Kbyte/s and ideal load of eNB to 500 Kbyte/s. Furthermore, the mobility model of the UE is stationary, and the initial position is fixed. We used no-control as a comparison target for the proposed method.

**Figure 5:**
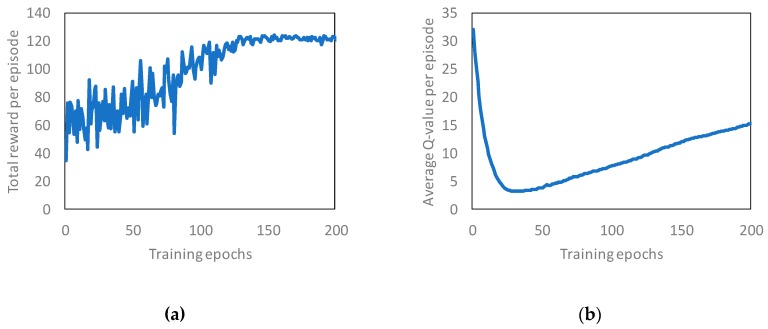
Learning result in basic evaluation: (**a**) total reward; (**b**) average q-value.

Figure 5 shows the training curves tracking the total reward and average q-value in the basic evaluation. We made the RL server learn the basic scenario until 200 epochs or approximately 40,000 data. One epoch means that the scenario for basic evaluation was executed once in the simulation. Based on Figure 5a, we confirmed that the total reward value increased as the number of epochs increased. At the same time, it was confirmed that the average Q-value also tends to converge. We think that the RL server learned the QoS allocation method for load balancing as the learning proceeds since the RL server gets a higher reward as the time to accommodate the current load in the ideal load $L_{ideal}$ increases. Based on Figure 5, we confirmed that the RL server learned the basic evaluation scenario. Thus, we evaluated the load balancing performance using the learned model.

**Figure 6:**
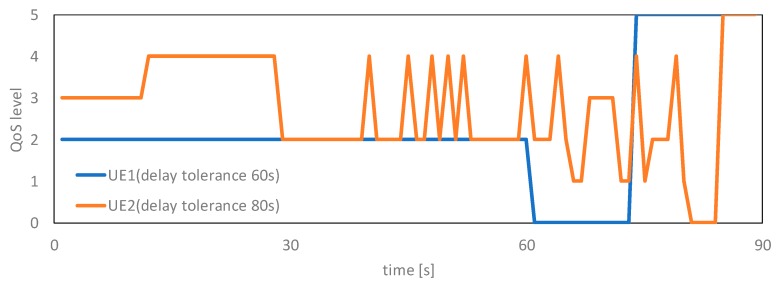
QoS level assignment.

Table 4 shows the excess amount from $L_{ideal}$ and the transmission amount sent within $L_{ideal}$, and Figure 6 presents the variation in the QoS level assigned to each UE from generation the content to data transmission completed. Table 4 confirmed that the proposed method reduced the excess data amount and increased the transmission amount sent as compared with the no-control case. In particular, we confirmed that the proposed method reduced the excess data amount by 78% and increased the transmission amount sent by 47% from the viewpoint of the bandwidth utilization efficiency. Furthermore, it can be observed from Figure 6 how the proposed method performed the QoS assignment. The QoS levels are high, middle, low, and empty in order from 0 to 4. The QoS level 5 indicates that the content has been completely sent. The proposed method assigned a high-priority QoS level to UE1 having content with a short delay tolerance from the generation of the content until 30 s. From this trend, we confirmed the tendency of the proposed method to determine the priority of the bandwidth allocation while considering a delay tolerance. Consequently, we confirmed that the proposed method can control the priority and balance the load while considering the delay tolerance in a scenario wherein the method of assigning priorities is clear. Moreover, we showed that an agent can learn the load balancing method while considering a delay tolerance by using the designed state, action, and reward. 

### 3.3. Performance Comparison

**Figure 7:**
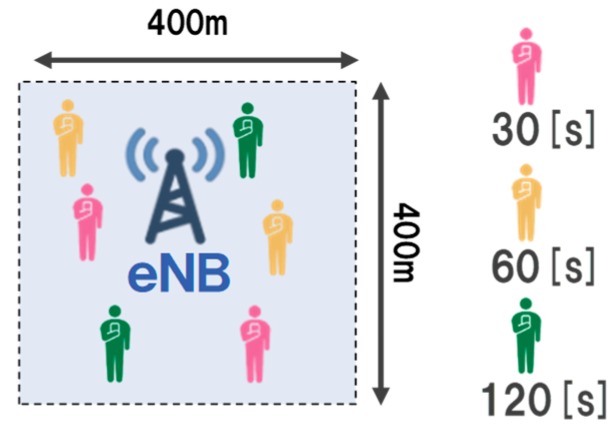
Topology of performance comparison.

We evaluated the cellular infrastructure utilization efficiency of the proposed method. In this evaluation, the proposed method, the timewise-offloading of MDOP, and no-control are compared. We created a scenario different from the basic evaluation for this evaluation. Table 5 shows the scenario used for comparing the performance, and Figure 7 presents the topology of the scenario for comparing the performance. We opine that multiple applications have a delay tolerance, such as local synchronization of map data on the cloud and life-logging data collected by wearable devices. We assumed that the UE is a smartphone or wearable device. Moreover, we assumed that there are four-types of delay tolerance contents from content-A to content-D in the mobile network. We created these contents according to [2,27]. The delay tolerance was provisionally set between 30 s to 120 s. In addition, we set the maximum transmission rate as 500 Kbyte/s. The UE’s mobility is stationary, and the initial position is determined based on a uniform distribution. The total mobile data traffic volume is 1,920 Mbytes in the evaluation environment. This mobile data traffic volume was 80% of the network topology capacity. We set the episode end time $t_{end}$ as 600 in Algorithm 1 as the simulation time is 600 s.

**Figure 8:**
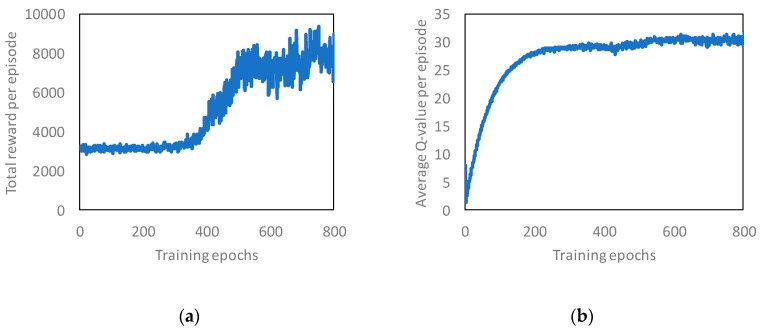
Learning result in performance comparison: (**a**) total reward; (**b**) average q-value.

Figure 8 shows the training curves tracking the total reward and average q-value. We made the RL server learn the performance comparison scenario until 800 epochs or approximately 9,600,000 data. One epoch means that the scenario for comparing performance was executed once in the simulation. Based on Figure 8a, we confirmed that the total reward value increased as the number of epochs increased. Similarly, we confirmed that the average Q-value also tends to converge. From Figure 8, we confirmed that the RL server learned the performance comparison scenario. Therefore, we evaluated the load balancing performance using the learned model as compared with the no-control case and the timewise-offloading of MDOP.

Table 6 shows the excess amount from $L_{ideal}$ and the transmission amount sent within $L_{ideal}$. As a result, the amount of traffic exceeds $L_{ideal}$ significantly with no-control and causes the occurrence of the temporal locality. The occurrence of the temporal locality degrades the quality of the communication because $L_{ideal}$ is set to avoid situations wherein the demand of the traffic causes packet loss. In contrast to no-control, the proposed method and the timewise-offloading of the MDOP reduce the traffic excess, and the proposed method reduces the traffic excess amount most. Furthermore, we confirmed that the proposed method reduced the excess data amount by 35% as compared with the time-wise offloading of MDOP. 

**Figure 9:**
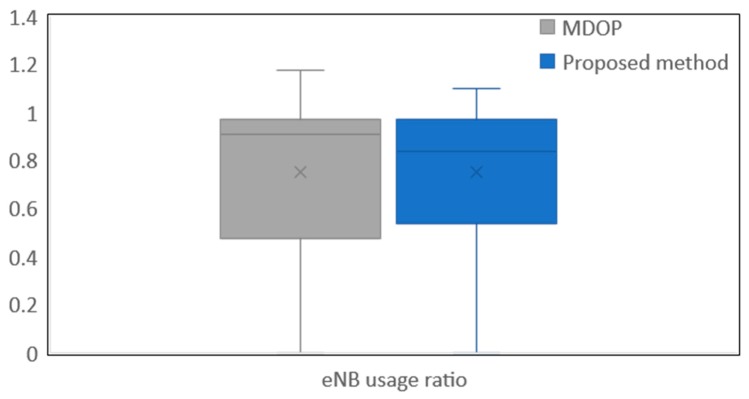
eNB usage ratio.

Secondly, we analyzed how the traffic exceeds $L_{ideal}$. Table 7 shows the details of the temporal locality. From Table 7, we can confirm that the amount of excessive data of the proposed method is smaller than the time-wise offloading of MDOP. Furthermore, the maximum excess time from $L_{ideal}$ is also shorter than the time-wise offloading of MDOP. In particular, from the viewpoint of the bandwidth utilization efficiency, it is desirable that the delay tolerance excess duration is short, because this value indicates that the temporal locality continues to occur within the excess duration. These results show that the proposed method suppresses the exceeded delay tolerance of content, because the MDOP sends the data at the maximum transmission rate without considering $L_{ideal}$ when the delay tolerance is exceeded. Thus, the more the proposed method suppresses the exceeded delay tolerance, the more the proposed method can suppress the occurrence of the temporal locality. However, the excess frequency from $L_{ideal}$ of the proposed method is greater than the time-wise offloading of MDOP. Although reducing the excess traffic and the excess time from $L_{ideal}$ is the most important factor in resolving the temporal locality, it is desirable that the excess frequency is small. In order to reduce the excess frequency from $L_{ideal}$, it appears to be necessary to consider the learning time increase and learning scenario diversification. By considering these, the proposed method more strictly controls the bandwidth. In addition to analyzing the excess traffic, we confirmed whether the traffic is distributed in terms of time. Figure 9 shows eNB usage rate based on $L_{ideal}$. As eNB usage ratio approaches 1.0, it indicates that the eNB’s performance is used sufficiently and temporal locality is reduced. From Figure 9, eNB usage ratio approached 1.0 when applying the proposed method for 600 s. This result shows that the proposed method can efficiently accommodate traffic to the eNB and fully utilizes the performance of the eNB. Therefore, we think that the proposed method balances traffic in terms of time. From Table 6, Table 7, and Figure 9, we confirmed that the proposed method can solve the temporal locality as compared with the time-wise offloading of MDOP.

**Figure 10:**
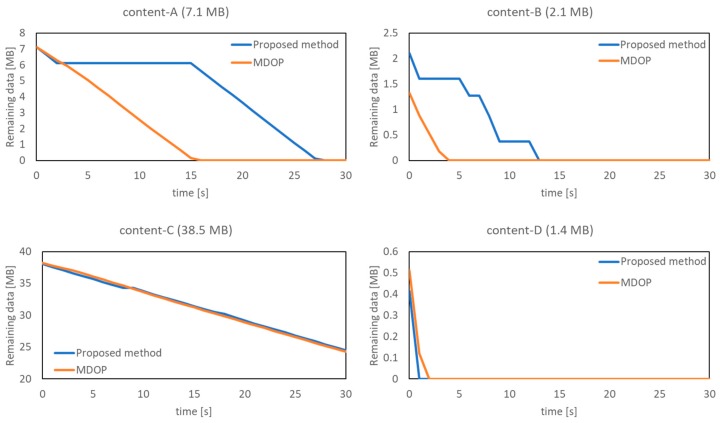
Trend of data remaining amount by contents.

Finally, we analyzed whether the proposed method allocates bandwidths to the UE while considering the delay tolerance. Table 8 shows the ratio of each data sent by the delay tolerance. This result shows that the proposed method improves the data transmission completion rate of the delay tolerance 60 s content as compared with the time-wise offloading of MDOP. In contrast, the transmission completion rate of the delay tolerance 120 s content is greater than the delay tolerance 30 s content with the use of the proposed method. Therefore, we focused on one UE from each content and confirmed the transition of the remaining data volume. Figure 10 shows the trend of the data remaining from content-A to content-D. Figure 10 shows the transition of the data remaining for each content from the generation of content-A with a delay tolerance of 30 s to the time at which the delay tolerance expires. This figure shows that the shorter the elapsed time, the content is sent faster, although shortened sent time is not the purpose of the proposed method. The purpose of the proposed method is to achieve time-wise balance the traffic. From Figure 10, it can be confirmed that the data transmission completion time of content-D is less than that obtained on using the MDOP when the proposed method is applied. Content-D has an allowable delay time of 120 s, and the content size is 1.4 MB, which is the smallest in this scenario. Furthermore, from the transition of the remaining content amount, it can be observed that as the content size is smaller, the proposed method preferentially completes the data transmission. Based on this trend, we can confirm that the proposed method completes the data transmission of the content of a small size as soon as possible and concentrates on the bandwidth allocation of the content of a large size. Among the four contents, the control of content-A was ideal because the bandwidth allocation was performed such that the UE transmits data by using a sufficient delay tolerance time. In this manner, we confirmed that the proposed method focuses on the content characteristics to control the transmission rate in order to solve the temporal locality. As our goal is to minimize the amount of excess data from $L_{ideal}$ and solve the temporal locality issue by controlling the bandwidth while taking into consideration the delay tolerance of the content and remaining content amount, this control is also appropriate in terms of load balancing. Based on Table 8 and Figure 10, we confirmed that the proposed method determined the priority and allocated the bandwidth based on the remaining content amount of the UE, delay tolerance time, and load on eNB.

Based on the above results, we confirmed that the proposed method improves the bandwidth utilization efficiency of cellular infrastructure by allocating bandwidth while taking into consideration the delay tolerance. In reinforcement learning, the reward function is an important factor for evaluating the action. In this paper, we applied only suppression of eNB load within $L_{ideal}$ as the reward function. We can expect that the performance will be further improved on examining the reward function.

## 4. Conclusions

We proposed a delay-tolerance-based mobile data offloading method using deep reinforcement learning. Principally, we applied deep reinforcement learning to the time-wise offloading of the MDOP to balance the load by taking into consideration the delay tolerance of the content. In our method, the RL server decides the priority of the bandwidth to be allocated to the UE based on the content delay tolerance, the remaining amount of content, and the load of eNB. The MDOP server then performs bandwidth control and load balancing. Initially, we evaluated whether the proposed method can assign a QoS level by considering the delay tolerance in a basic evaluation. The obtained result demonstrated that the proposed method can learn how to allocate the QoS level for balancing the load. Moreover, we compared the cellular infrastructure bandwidth utilization efficiency of the proposed method, the time-wise offloading of MDOP, and no-control. As a result of the evaluation based on the assumption that there are four types of contents with different delay tolerances, the proposed method decreased the traffic in excess of the control target value by 35% as compared with the time-wise offloading of MDOP. Furthermore, on the basis of the maximum excess traffic amount and the maximum excess time, we confirmed that the proposed method further resolves the temporal locality. Moreover, the proposed method increased the ratio of data sent by the delay tolerance as compared with the time-wise offloading of MDOP. Therefore, we opine that the cellular bandwidth utilization efficiency has been improved by allocating the bandwidth while taking into consideration the delay tolerance and load balancing. Although we show the effectiveness of the proposed method about load balancing by focusing on delay tolerance, we need to consider the installation location of MDOP server and RL server and environment information collection method in order to apply the proposed method to a real environment. Furthermore, the current learning model focuses on UE present in one eNB, and it is not easy to use the current model in the situation where there are multiple eNBs to be considered in a real environment. In the future, we will evaluate and consider our method in multiple eNBs, reduce learning cost, and consider the proposed method’s design to apply to a real environment. Moreover, we intend to evaluate the mobility model of UE using vehicles and the traffic model, including various delay tolerances to demonstrate the effects of the proposed method in a real environment.

## Figures and Tables

**Table 1:** Learning parameters of the proposed method at t.

Value	Attribute
Remaining amount of content $\left(content_{ra}\right)$	UE information
Delay tolerance time of content $\left(content_{dt}\right)$	UE information
Maximum $content_{ra}$ of UEs	Relative information
Median $content_{ra}$ of UEs	Relative information
Minimum $content_{ra}$ of UEs	Relative information
Maximum $content_{dt}$ of UEs	Relative information
Median $content_{dt}$ of UEs	Relative information
Minimum $content_{dt}$ of UEs	Relative information
Available bandwidth	eNB information
Current load	eNB information
Ideal load	eNB information
Current time	eNB information

**Table 2:** Network environment model.

Parameters	Value
Transmission power of UE	23 [dBm]
Transmission power of eNB	46 [dBm]
Cellular bandwidth	10 [MHz]
Frequency	2.0 [GHz]
Antenna	Omnidirectional antenna
TCP	New Reno

**Table 3:** Scenario for basic evaluation.

Parameters	Value	
	UE1	UE2
Simulation time	100 [s]	
Number of eNB	1	
Maximum data reception amount of eNB	625 [Kbyte/s]	
Ideal load	500 [Kbyte/s]	
Number of UE	2	
Max send rate of UE	1000 [Kbyte/s]	
Movement method	Stationary	
Initial position of UE	Fixed	
Data generation interval	100 [s]	
Content size	30 [MB]	10 [MB]
Delay tolerance of the content	60 [s]	80 [s]

**Table 4:** Excess volume and transmission volume for basic evaluation.

	$\mathrm{Excess}\;\mathrm{Amount}\;\mathrm{from}\;L_{ideal}\;[\mathrm{MB}]$	$\mathrm{Transmission}\;\mathrm{Amount}\;\mathrm{Sent}\;\mathrm{within}\;L_{ideal}\;[\mathrm{MB}]$
No-control	24.7	21.4
Proposed method	5.4	40.6

**Table 5:** Scenario for comparing performance.

Parameters	Value			
	content-A	content-B	content-C	content-D
Simulation time	600 [s]			
Number of eNB	1			
Maximum data reception amount of eNB	5 [Mbyte/s]			
Ideal load	4 [Mbyte/s]			
Maximum send rate of UE	500 [Kbyte/s]			
Number of UE	6	4	7	3
Movement method	Stationary			
Initial position of UE	Uniform distribution			
Content size	7.1 [MB]	2.1 [MB]	38.5 [MB]	1.4 [MB]
Data generation interval	30 [s]	60 [s]	60 [s]	120 [s]
Delay tolerance of content	30 [s]	60 [s]	60 [s]	120 [s]

**Table 6:** Excess volume and transmission volume for performance comparison.

	$\mathrm{Excess}\;\mathrm{Amount}\;\mathrm{from}\;L_{ideal}\;[\mathrm{MB}]$	$\mathrm{Transmission}\;\mathrm{Amount}\;\mathrm{Sent}\;\mathrm{within}\;L_{ideal}\;[\mathrm{MB}]$
No-control	33.9	1769.2
MDOP	4.2	1795.0
Proposed method	2.7	1797.0

**Table 7:** Details of temporal locality.

	$\mathrm{Maximum}\;\mathrm{Excess}\;\mathrm{Traffic}\;\mathrm{Amount}\;\mathrm{from}\;L_{ideal}\;[\mathrm{MB}]\;$	$\mathrm{Maximum}\;\mathrm{Excess}\;\mathrm{Time}\;\mathrm{from}\;L_{ideal}\;[s]$	$\mathrm{Excess}\;\mathrm{Frequency}\;\mathrm{from}\;L_{ideal}\;[\mathrm{times}]$
MDOP	0.7	4	15
Proposed method	0.4	2	16

**Table 8:** Ratio of data sent by delay tolerance.

Delay Tolerance [s]	MDOP	Proposed Method
30	0.95	0.95
60	0.89	0.91
120	1.0	1.0
